# Association between triglyceride-glucose index trajectories and carotid atherosclerosis progression

**DOI:** 10.1186/s12933-023-01847-y

**Published:** 2023-05-31

**Authors:** Haixu Yu, Liyuan Tao, Yan-Guang Li, Lincheng Yang, Dan Liu, Yang Wang, Xiaoyan Hao, Honghai He, Ying Che, Peng Wang, Wei Zhao, Wei Gao

**Affiliations:** 1 NHC Key Laboratory of Cardiovascular Molecular Biology and Regulatory Peptides, Key Laboratory of Molecular Cardiovascular Science, Beijing Key Laboratory of Cardiovascular Receptors Research, Department of Cardiology, Institute of Vascular Medicine, Ministry of Education, Peking University Third Hospital, 100191 Beijing, China; 2 Research Center of Clinical Epidemiology, Peking University Third Hospital, Beijing, 100191 China; 3 Physical Examination Center, Peking University Third Hospital, Beijing, 100191 China

**Keywords:** Triglyceride-glucose index, Insulin resistance, Carotid atherosclerosis, Progression, Longitudinal study

## Abstract

**Background:**

The triglyceride-glucose (TyG) index has been recognized as being an alternative cardiometabolic biomarker for insulin resistance associated with the development and prognosis of cardiovascular disease (CVD). However, the prospective relationship between baseline and long-term trajectories of the TyG index and carotid atherosclerosis (CAS) progression has yet to be investigated.

**Methods:**

This longitudinal prospective cohort study included 10,380 adults with multiple general health checks at Peking University Third Hospital from January 2011 to December 2020. The TyG index was calculated as ln (fasting triglyceride [mg/dL] × fasting glucose [mg/dL]/2). The latent class trajectory modeling method was used to analyze the TyG index trajectories over the follow-up. Based on univariate and multivariate Cox proportional hazards analyses, hazard ratios (HRs) and 95% confidence intervals (CIs) were calculated for the baseline and trajectory of the TyG index.

****Results**:**

During a median follow-up period of 757 days, 1813 participants developed CAS progression. Each 1-standard deviation (SD) increase in the TyG index was associated with a 7% higher risk of CAS progression after adjusting for traditional CVD risk factors (HR = 1.067, 95% CI 1.006–1.132). Similar results were observed when the TyG index was expressed as quartiles. According to different trajectory patterns, participants were categorized into low-stable, moderate-stable, and high-increasing groups. After multivariate adjustment, the moderate-stable group had a 1.139-fold (95% CI 1.021–1.272) risk of CAS progression. The high-increasing trajectory of the TyG index tended to be associated with CAS progression (HR = 1.206, 95% CI 0.961–1.513).

****Conclusions**:**

Participants with higher baseline and moderate-stable trajectory of the TyG index were associated with CAS progression. Long-term trajectories of the TyG index can help to identify individuals at a higher risk of CAS progression who deserve specific preventive and therapeutic approaches.

## Introduction

Cardiovascular disease (CVD) is the leading cause of disability and premature mortality throughout the world, with a substantial economic and health burden. According to the Global Burden of Disease Study 2019, the number of prevalent cases of total CVD nearly doubled from 271 million in 1990 to 523 million in 2019 [1]. As the highest number of CVD deaths in countries throughout the world, CVD in China is challenged by the multiple pressures of population aging and the constant increase in the prevalence of cardiometabolic risk factors, including hypertension, obesity, diabetes, and metabolic syndrome [2]. Long-term exposure to cardiometabolic risk factors predisposes atherosclerotic plaque formation and development, thus allowing for the progression from subclinical stages to clinical manifestations.

A previous report suggests that stroke has caused the highest number of deaths in China, with 2.2 million deaths reported in 2019 [2]. Carotid atherosclerosis (CAS) is the most common cause of ischemic stroke, where it accounts for approximately 18–25% of stroke deaths [3, 4]. In global individuals aged 30–79 years in 2020, the prevalence of carotid plaque was estimated to be 21.1%, and carotid intima-media thickness was estimated to be 27.6% [5]. In China, the prevalence of CAS was 27.22% in people aged 30–79 years [6]. Thus, the considerable disease burden of CAS has resulted in efforts on effective preventive health strategies and early assessment, especially for CAS progression.

As the pathophysiological hallmark of obesity, metabolic syndrome, and diabetes, insulin resistance (which is characterized by the attenuated insulin sensitivity of peripheral tissues) leads to oxidative stress, mild inflammatory responses, and endothelial dysfunction, which contributes to atherosclerosis progression [7]. As a surrogate marker for the clinical evaluation of insulin resistance, the triglyceride-glucose (TyG) index, which is calculated by fasting triglycerides (TG) and fasting blood glucose (FBG), is more reliable than the common homeostatic model of insulin resistance (HOMA-IR) [8]. The TyG index is significantly correlated with insulin resistance as measured by the hyperinsulinemic-euglycemic clamp (which is the gold standard for assessing insulin resistance) [9]. Previous studies have suggested that the TyG index is associated with morbidity and mortality of CVD, the progression of coronary artery calcification (which is an established marker of subclinical atherosclerosis), and arterial stiffness [10–13]. However, most prior cross-sectional studies have focused on elevated baseline levels rather than dynamic changes over time. Although previous studies have reported on the relationship between the TyG index and CAS [14–16], the association between the TyG index and CAS progression and its trajectory derived from multiple measurements over time has not been performed.

Therefore, we hypothesized that dynamic changes in insulin resistance may modify the progression of CAS. Our study aimed to explore the associations between baseline levels and the trajectory of the TyG index and CAS progression through a single-center, large longitudinal cohort study of the Chinese population.

## Methods

### Study Design and setting

**Fig. 1:**
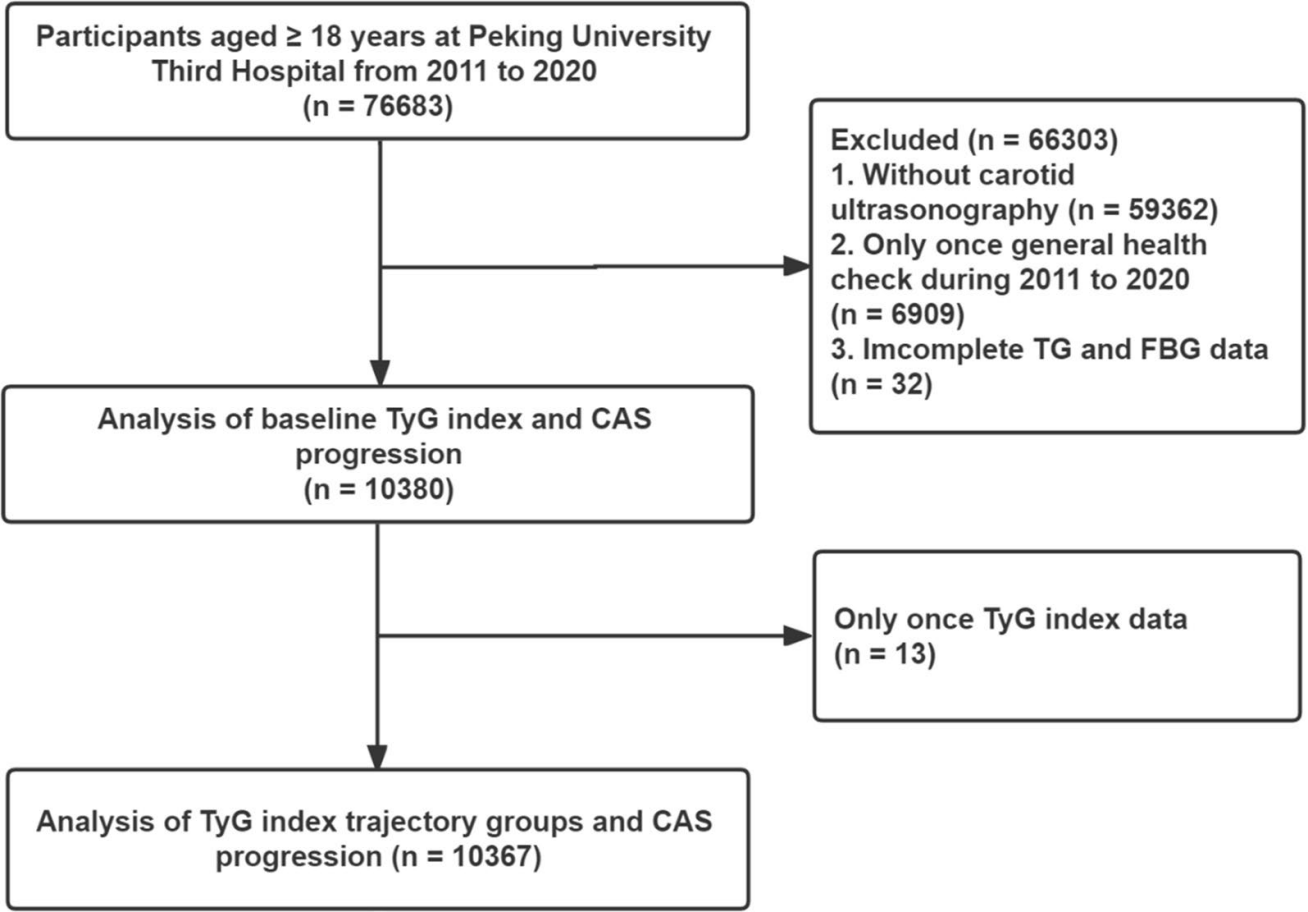
Flowchart of study participants. TyG index, triglyceride-glucose index; CAS, carotid atherosclerosis; FBG, fasting blood glucose; TG, triglyceride

This prospective, population-based, longitudinal cohort study was conducted from general health checks at Peking University Third Hospital. From January 2011 to December 2020, a total of 76,683 participants ≥ 18 years who underwent general medical examinations were enrolled [17]. The exclusion criteria were as follows: (1) no carotid ultrasonography (n = 59,362); (2) only one general health check (n = 6969); (3) incomplete TG and FBG data (n = 32); and (4) only one TyG index data point. A total of 10,380 participants were included in the baseline data analysis. For the trajectory analysis, 10,367 participants were included. The research flowchart is shown in Fig. 1. This study was conducted in accordance with the Declaration of Helsinki and approved by the Peking University Third Hospital Medical Science Research Ethics Committee (M2021098). All of the patients provided written informed consent.

### Characteristics and definition

Trained interviewers collected information by using a standardized questionnaire, including demographic characteristics and medical history. Body mass index (BMI) was calculated as weight (kg)/height (m)^2^. In addition, systolic blood pressure (SBP) and diastolic blood pressure (DBP) were recorded as the average of three measurements in the seated position by using an automatic blood pressure monitor. Hypertension was defined as SBP ≥ 140 mmHg or DBP ≥ 90 mmHg, the current use of antihypertensive medications, or a self-reported history of hypertension. Diabetes was defined as fasting blood glucose (FBG) ≥ 7.0 mmol/L in the cohort exam or self-reported history of diabetes as diagnosed by physicians [15]. Moreover, the examined biochemical parameters included total cholesterol (TC), low-density lipoprotein cholesterol (LDL-C), triglycerides (TG), high-density lipoprotein cholesterol (HDL-C), and FBG. The TyG index was calculated as ln (fasting triglyceride [mg/dL]× FBG [mg/dL]/2), and TyG-BMI was computed by using TyG index × BMI [8]. Peripheral blood samples were tested by the Clinical Laboratory Department of Peking University Third Hospital with a laboratory accreditation certificate. Fasting blood samples were collected in the morning, and a range of biochemical parameters were analyzed by using a commercially available assay kit on the Beckman Coulter platform (Beckman Coulter Inc., Brea, CA, USA) clinical chemistry analyzer.

### Carotid ultrasonography and study outcome

The ultrasonographic assessments of bilateral carotid arteries were manually performed by experienced and certified doctors from the Ultrasound Department at Peking University Third Hospital who were blinded to this study. The participants were examined by using a GE® Vivid i/E95 high-resolution ultrasound system (GE Healthcare, Milwaukee, WI, USA) and a 7.5–12 MHz phased array probe. Abnormal cIMT was defined as a maximum cIMT value ≥ 0.9 mm, which was the maximum distance between the interface of the lumen-intima and media-adventitia. Carotid plaque was defined as cIMT ≥ 1.5 mm, a focal structure protruding into the arterial lumen ≥ 0.5 mm, or ≥ 50% of the surrounding cIMT value. In addition, carotid atherosclerosis (CAS) progression was defined as the appearance of newly developed carotid stenosis, carotid plaque, or cIMT during follow-up compared with baseline. For individuals with combined carotid plaque and cIMT, baseline data and follow-up outcomes were defined according to superior manifestations (i.e., carotid plaques) [18].

### Statistical analysis

According to the quartile groups of the baseline TyG index in the longitudinal analysis, descriptive characteristics were summarized as the mean ± standard deviation, medians with interquartile ranges (IQRs), and frequencies with percentages (%), when applicable. Comparisons of continuous variables were analyzed by using Mann‒Whitney *U* tests or Kruskal‒Wallis *H*-tests (two or more independent samples), and comparisons of categorical variables were analyzed by using the chi-squared test or Fisher’s exact test. The Cox proportional hazards regression model was utilized to analyze the relationship between baseline TyG index quartiles and TyG index per SD change and CAS progression. Multiple potential confounders in this study were also considered, including age, male sex, BMI, hypertension, and diabetes. Using the log-rank test, the cumulative incidence of CAS progression was computed via the TyG index quartiles by using the Kaplan‒Meier survival curve.

A latent class trajectory modeling (LCTM) approach was used to analyze the long-term trajectory of the TyG index. This specialized form of finite mixture modeling allowed us to simplify the heterogeneous longitudinal course of the TyG index into homogeneous classes and to investigate latent classes of participants following similar trajectories over time. Models were fitted by using the *lcmm* package (version 1.9.5) in R (version 4.2.0, Vienna, Austria). We determined the optimal number of classes by combining the following criteria: (1) the lowest Bayesian Information Criteria (BIC) while maintaining clinical relevance and parsimony of the model; (2) the average probability of assignments above 70% for all of the latent classes; and (3) the proportion of individuals estimated to be assigned to each class size being greater than 2% [19]. The characteristics of the TyG index trajectory were compared (as appropriate) by using ANOVA or Kruskal‒Wallis *H*-tests for continuous variables and chi-squared tests for categorical variables. To investigate the association between trajectory classes and CAS progression, a Cox proportional hazards regression model was used with follow-up time as the time scale.

All of the statistical analyses were performed by using IBM SPSS software (version 23.0, SPSS Inc., Chicago, IL), GraphPad Prism (version 7.0, La Jolla, CA), and R (version 4.2.0) with RStudio (version 2022.02.3, Boston, MA) and associated packages. Two-tailed P values < 0.05 were considered to be statistically significant.

## Results

### Baseline characteristics according to quartiles of TyG index

In the study of 10,380 eligible participants, the median age was 50 (40–61) years, 5421 (52.2%) participants were males, and the median TyG index was 8.7 (8.3–9.1). During the median follow-up of 757 days (IQR: 388–844 days), 1815 (17.5%) patients reached the study outcome. In accordance with the TyG index level, participants were stratified into four groups (Table 1). Higher quartiles of the TyG index were more likely to be older, male, have a higher BMI, and have a higher prevalence of hypertension and diabetes compared to those in the lowest quartile. Furthermore, SBP, DBP, FBG, TC, TG, LDL-C, TyG index, and TyG-BMI index were all positively correlated with increasing TyG index quartiles, whereas HDL-C levels were negatively correlated (all *P* for trend < 0.001). Based on the findings of this study, a higher TyG index was associated with increased cardiometabolic risk factors among the participants.

**Table 1:** Baseline characteristics of study participants according to quartiles of TyG index

Characteristics	Quartiles of TyG index				*P* for trend
	Q1 (6.82–8.27)	Q2 (8.27–8.66)	Q3 (8.66–9.08)	Q4 (9.08–12.89)	
n	2601	2592	2593	2594	
Age, years	45 (34–54)	50 (40–62)	53 (43–63)	54 (46–64)	< 0.001
Male, n (%)	1023 (39.4%)	1240 (23.0%)	1476 (27.4%)	1657 (30.7%)	< 0.001
BMI, kg/m^2^	22.3 (20.5–24.4)	24.0 (22.0-26.1)	25.1 (23.2–27.2)	26.2 (24.2–28.3)	< 0.001
Hypertension, n (%)	165 (6.4%)	323 (12.5%)	481 (18.5%)	610 (23.7%)	< 0.001
Diabetes, n (%)	51 (2.0%)	125 (4.8%)	277 (10.7%)	733 (28.4%)	< 0.001
SBP, mmHg	118 (107–130)	124 (114–137)	130 (119–141)	135 (123–147)	< 0.001
DBP, mmHg	72 (65–80)	75 (69–83)	79 (71–85)	82 (75–88)	< 0.001
FBG, mmol/L	4.9 (4.6–5.2)	5.1 (4.8–5.5)	5.3 (5.0-5.8)	5.7 (5.2–6.8)	< 0.001
TC, mmol/L	4.4 (3.9-5.0)	4.8 (4.2–5.4)	5.0 (4.4–5.5)	5.1 (4.5–5.8)	< 0.001
TG, mmol/L	0.8 (0.7–0.9)	1.2 (1.1–1.3)	1.6 (1.5–1.8)	2.6 (2.2–3.3)	< 0.001
LDL-C, mmol/L	2.7 (2.3–3.2)	3.1 (2.6–3.6)	3.3 (2.7–3.8)	3.2 (2.6–3.8)	< 0.001
HDL-C, mmol/L	1.5 (1.3–1.7)	1.3 (1.2–1.5)	1.2 (1.1–1.4)	1.1 (1.0-1.2)	< 0.001
TyG index	8.0 (7.8–8.2)	8.5 (8.4–8.6)	8.9 (8.8-9.0)	9.4 (9.2–9.7)	< 0.001
TyG-BMI index	177.4 (162.3-195.5)	202.8 (185.3-221.1)	222.2 (205.0-241.3)	248.6 (228.0-271.7)	< 0.001
CAS progression, n (%)	383 (14.7%)	466 (18.0%)	464 (17.9%)	500 (19.3%)	< 0.001

TyG index, triglyceride-glucose index; BMI, body mass index; SBP, systolic blood pressure; DBP, diastolic blood pressure; FBG, fasting blood glucose; TC, total cholesterol; TG, triglyceride; LDL-C, low-density lipoprotein cholesterol; HDL-C, high-density lipoprotein cholesterol

### Associations between baseline TyG index and CAS progression

**Fig. 2:**
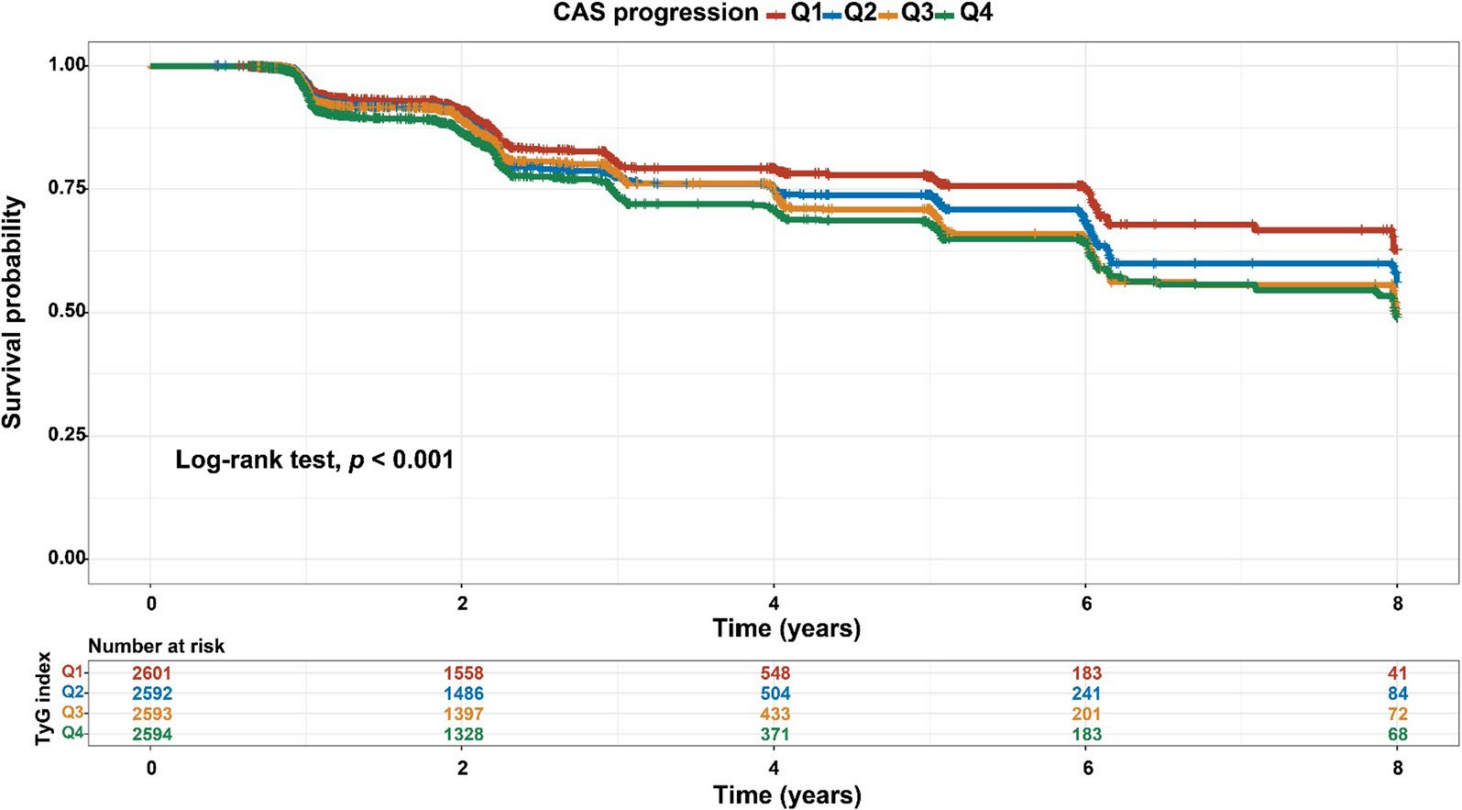
Kaplan‒Meier survival analysis curves for CAS progression based on quartiles of baseline TyG index. TyG index: Q1 (6.82–8.27), Q2 (8.27–8.66), Q3 (8.66–9.08), and Q4 (9.08–12.89). CAS, carotid atherosclerosis; TyG index, triglyceride-glucose index

As shown in Table 1, the risk of progression of CAS increased with increasing TyG quartiles. After full adjustment for potential confounders, a 1-SD increase in the TyG index was associated with a 7% increased risk of CAS progression in the multivariate model that measured the TyG index as a continuous variable (HR = 1.067, 95% CI 1.006–1.132, *P* = 0.032, as shown in Table 2). Similar results were observed when individuals were categorized by TyG index quartiles; specifically, the highest risk of CAS progression was observed among participants with the highest TyG index quartile in three different adjusted models (all *P <* 0.05, Table 2). As a result of the final model, the HRs with 95% CIs for CAS progression comparing the second, third, and fourth quartiles of the TyG index with the first quartile were 1.087 (95% CI 0.944–1.252), 1.001 (95% CI 0.839–1.194), and 1.178 (95% CI 1.011–1.372), respectively (Table 2). Based on the quartiles of the baseline TyG index, Fig. 2 illustrates the Kaplan‒Meier survival curves for CAS progression (log-rank test, *P* < 0.001). These findings suggested that the baseline TyG index was significantly correlated with CAS progression.

**Table 2:** Hazard ratios (95% confidence intervals) of CAS progression by baseline TyG index

TyG index	CAS progression (N)	Unadjusted HR (95% CI)	*P* value	Model 1 HR (95% CI)	*P* value	Model 2 HR (95% CI)	*P* value
Quartile 1	383/2601	Reference		Reference		Reference	
Quartile 2	466/2592	1.193 (1.042–1.366)	0.011	1.120 (0.977–1.285)	0.104	1.087 (0.944–1.252)	0.246
Quartile 3	464/2593	1.290 (1.126–1.477)	< 0.001	1.176 (1.024–1.351)	0.022	1.001 (0.839–1.194)	0.126
Quartile 4	500/2594	1.440 (1.260–1.646)	< 0.001	1.281 (1.116–1.472)	< 0.001	1.178 (1.011–1.372)	0.036
Per 1 SD	1813/10,380	1.150 (1.094–1.210)	< 0.001	1.099 (1.042–1.159)	< 0.001	1.067 (1.006–1.132)	0.032

Model 1: Adjusted for age and male; Model 2: Adjusted for age, male, BMI, hypertension, and diabetes.

CAS, carotid atherosclerosis progression; TyG index, triglyceride-glucose index; HR, hazard ratio; CI, confidence interval; SD, standard deviation

### Baseline characteristics according to TyG index trajectories

**Fig. 3:**
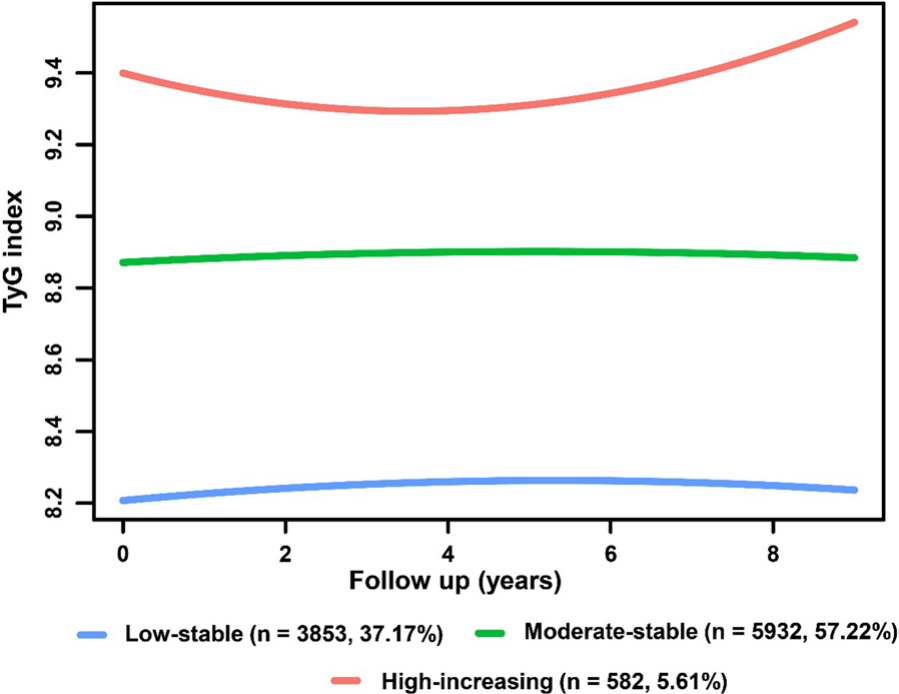
TyG index trajectory groups and percentage of the participants in the group. TyG index, triglyceride-glucose index

**Fig. 4:**
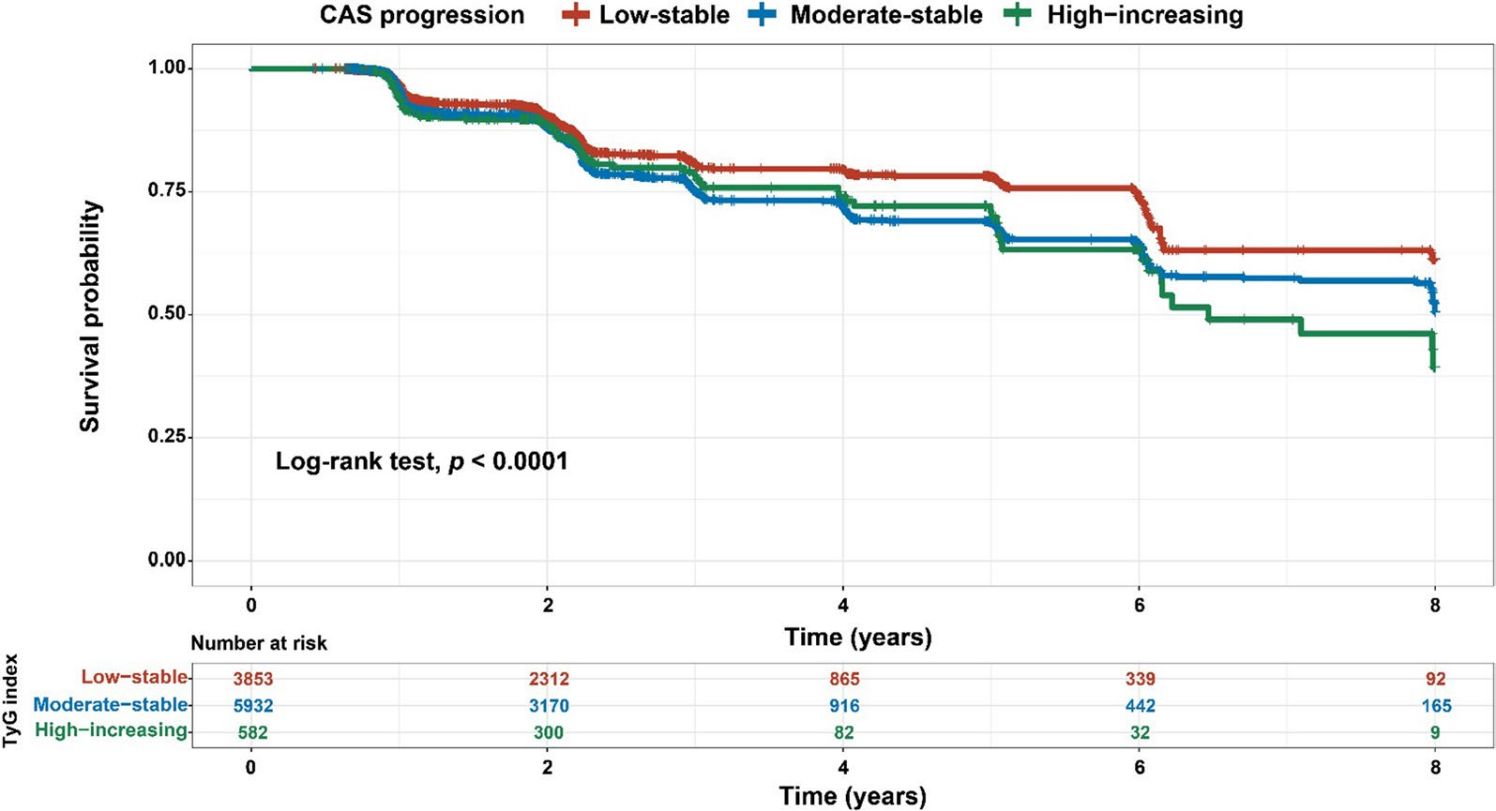
Kaplan‒Meier survival analysis curves for CAS progression based on trajectories of the baseline TyG index. CAS, carotid atherosclerosis; TyG index, triglyceride-glucose index

During the trajectory analysis, a total of 10,367 participants were included (Fig. 1). Following the application of model-adequacy criteria and the rule of interpretability, three distinct trajectories of the TyG index were selected. In Fig. 3, we show the final latent class trajectory models for the low-stable (n = 3853), moderate-stable (n = 5932), and high-increasing TyG index trajectory groups (n = 582). Table 3 summarizes the baseline demographics and clinical characteristics of the TyG index trajectories. Higher levels of TyG index trajectories were more likely to be older and male, as well as have hypertension, diabetes, and higher levels of BMI, blood pressure, baseline lipid profiles (TC, TG, and LDL-C), FBG, TyG index, and TyG-BMI index (all *P* for trend < 0.001). In accordance with the baseline quartiles of the TyG index, higher levels of the TyG index trajectory were associated with increased cardiometabolic risk factors. As TyG index trajectories increased, the risk of the progression of CAS increased (Table 3). The results indicated that the TyG index trajectory was significantly correlated with the progression of CAS. Furthermore, the Kaplan‒Meier survival curves for CAS progression (log-rank test, *P* < 0.001) are presented in Fig. 4.

**Table 3:** Baseline characteristics of study participants according to trajectories of the TyG index

Characteristics	Low-stable (n = 3853)	Moderate-stable (n = 5932)	High-increasing (n = 582)	*P* for trend
Age, years	46 (35–55)	54 (44–63)	54 (46–62)	< 0.001
Male, n (%)	1603 (41.6%)	3381 (57.0%)	416 (71.5%)	< 0.001
BMI, kg/m^2^	22.7 (20.8–24.8)	25.2 (23.3–27.4)	26.5 (24.8–28.4)	< 0.001
Hypertension, n (%)	284 (7.4%)	1139 (19.2%)	154 (26.5%)	< 0.001
Diabetes, n (%)	92 (2.4%)	814 (13.7%)	279 (47.9%)	< 0.001
SBP, mmHg	120 (109–131)	130 (119–143)	136 (125–148)	< 0.001
DBP, mmHg	73 (66–80)	79 (71–86)	84 (77–90)	< 0.001
FBG, mmol/L	4.9 (4.7–5.3)	5.3 (5.0-5.9)	6.5 (5.4–8.9)	< 0.001
TC, mmol/L	4.5 (4.0-5.1)	5.0 (4.4–5.6)	5.3 (4.6-6.0)	< 0.001
TG, mmol/L	0.9 (0.7–1.1)	1.7 (1.3–2.2)	4.0 (3.0-5.4)	< 0.001
LDL-C, mmol/L	2.8 (2.3–3.3)	3.2 (2.7–3.8)	2.9 (2.2–3.6)	< 0.001
HDL-C, mmol/L	1.4 (1.2–1.7)	1.2 (1.0-1.4)	1.0 (0.9–1.2)	< 0.001
TyG index	8.2 (8.0-8.4)	8.9 (8.7–9.2)	10.0 (9.7–10.3)	< 0.001
TyG-BMI index	184.3 (167.2-203.6)	224.8 (205.5-247.3)	266.5 (247.0-290.9)	< 0.001
CAS progression, n (%)	608 (15.8%)	1099 (18.5%)	106 (18.2%)	0.002

TyG index, triglyceride-glucose index; BMI, body mass index; SBP, systolic blood pressure; DBP, diastolic blood pressure; FBG, fasting blood glucose; TC, total cholesterol; TG, triglyceride; LDL-C, low-density lipoprotein cholesterol; HDL-C, high-density lipoprotein cholesterol

### Associations between TyG index trajectories and CAS progression

The relationships between TyG index trajectories and CAS progression are presented in Table 4. Compared with the low-stable group, the moderate-stable group and the high-increasing group were independently associated with CAS progression. After adjusting for age, male sex, BMI, hypertension, and diabetes covariates, the moderate-stable group had a 1.139-fold (95% CI 1.021–1.272) risk of CAS progression. Moreover, there was no significant correlation between the high-increasing group and the study outcomes (HR = 1.206, 95% CI 0.961–1.513, *P* = 0.105).

**Table 4:** Hazard ratios (95% confidence intervals) of CAS progression by trajectory groups of TyG index

TyG index trajectories	CAS progression (N)	Unadjusted HR (95% CI)	*P* value	Model 1 HR (95% CI)	*P* value	Model 2 HR (95% CI)	*P* value
Low-stable	608/3853	Reference		Reference		Reference	
Moderate-stable	1,099/5,932	1.306 (1.182–1.442)	< 0.001	1.201 (1.084–1.330)	< 0.001	1.144 (1.025–1.276)	0.016
High-increasing	106/582	1.388 (1.129–1.707)	0.002	1.254 (1.017–1.546)	0.034	1.208 (0.971–1.502)	0.090

Model 1: Adjusted for age and male; Model 2: Adjusted for age, male, BMI, hypertension, and diabetes. Abbreviations: CAS, carotid atherosclerosis progression; HR, hazard ratio; LDL-C, low-density lipoprotein cholesterol; HDL-C, high-density lipoprotein cholesterol

## Discussion

In this large longitudinal cohort study derived from general health check-ups, we first examined the relationship between baseline and longitudinal trajectories of the TyG index and CAS progression. A higher baseline TyG index was significantly associated with CAS progression in both continuous and quartile forms. Furthermore, we identified three distinct trajectories of the TyG index conferring a different risk of CAS progression, including the low-stable, moderate-stable, and high-increasing trajectories. The prognostic value of the longitudinal moderate-stable trajectory of the TyG index in CAS progression was independent of the baseline TyG index. Moreover, the high-increasing trajectory of the TyG index tended to be associated with CAS progression. These results suggest a pathophysiological mechanism for continuously higher levels of insulin resistance in the pathogenesis of CAS progression.

The TyG index has been extensively studied in the field of atherosclerotic cardiovascular disease (ASCVD) in recent years, as well as coronary artery calcification [20]. These various studies have suggested that the TyG index is independently associated with the incidence, development, progression, and adverse events of ASCVD. The results of the baseline TyG index and CAS progression in our study were consistent with the current knowledge that has been previously reported. Li et al. demonstrated a positive association between the TyG index and carotid atherosclerosis. The association was shown to be higher in males and middle-aged individuals than in females and elderly individuals [16]. Additionally, Zhang et al. detected that the TyG index can be used as a dose-responsive indicator of carotid plaques in one longitudinal study containing 2370 subjects [14]. Wang et al. found that the TyG index was associated with increased odds of atherosclerosis in coronary, intracranial, and extracranial arteries, and the TyG index exhibited better net reclassification improvement ability than HOMA-IR for intracranial atherosclerotic plaques [21]. In the first part of this study, similar results were observed for the association between the baseline TyG index and CAS progression. However, most of the previous studies focused on the baseline or single level of the TyG index, and there were limited data on the dynamic changes over time. Although the study demonstrated insufficient statistical significance in the multivariate adjustment analysis, the results in the high-increasing group still demonstrated an increasing trend compared with the other groups. It may be relevant to examine populations that originate from health checkups rather than ASCVD, which resulted in a wider confidence interval among the high-increasing groups. In the field of studies on the trajectory of the TyG index, little attention has been given to the progression of carotid atherosclerosis. Gao et al. demonstrated that a higher TyG index is independently associated with incident peripheral artery disease. Trajectories denoting long-term exposure to a higher TyG index provide a cumulative burden of risk for future peripheral artery disease [22]. Yan et al. found that elevated levels of the baseline TyG index and a higher long-term trajectory of the TyG index were independently associated with increased arterial stiffness [23]. All of these studies indicated a previously overlooked issue of the uniqueness of TyG trajectory characteristics in the study of ASCVD. Our study determined the influence of the baseline TyG index and different dynamics on CAS progression. The results support that long-term trajectories of the TyG index may help to identify individuals at higher risks of CAS progression who deserve specific prevention and treatment.

The available methods for directly measuring insulin resistance are invasive, complex, and costly. The gold standard of the hyperinsulinemic-euglycemic clamp has correspondingly been substituted by several surrogate markers, including traditional HOMA-IR and the latest TyG index [9]. Therefore, the TyG index was used as a biomarker of insulin resistance in this large-scale, community-based prospective cohort study. However, the specific mechanism underlying the relationship between the TyG index and atherosclerosis progression remains to be explained. Numerous studies have identified potential mechanisms underlying the association between insulin resistance and cardiometabolic disorders (such as ASCVD). It is obvious that the TyG index consists of two important components: lipid-related and glucose-related processes. Due to glucose metabolism imbalance and systemic lipid disturbances, insulin resistance mediates systemic inflammation and vascular remodeling by promoting endothelial dysfunction and oxidative stress, which causes the initiation of atherosclerosis [24–27]. Therefore, insulin resistance drives the development of atherogenic dyslipidemia in a low-grade inflammatory state and subsequently promotes hypercoagulability and atherosclerosis [28]. Additionally, insulin resistance is related to arterial stiffness and coronary artery calcification through effects on platelet adhesion, activation, and aggregation [29, 30]. Furthermore, insulin resistance may promote excessive glycosylation, smooth muscle cell proliferation, and collagen deposition due to the presence of hyperglycemia [7]. It can be concluded that the presence of metabolic abnormalities, inflammatory oxidative stress, endothelial dysfunction, and smooth muscle cell dysfunction in participants with a continuously higher level of insulin resistance is responsible for the pathophysiological progression of atherosclerosis [20].

### Strengths and limitations

The main advantage of this study was the large, longitudinal population cohort with repeated measurements of TyG index profiles and carotid ultrasound results. This prospective study extended the investigation to determine the baseline and follow-up relationship between the TyG index and carotid atherosclerosis progression. The longitudinal lipid trajectory method was developed by using LCTM, which provided detailed insight into lipid trajectory changes over time. Moreover, there were several limitations to our study that should be noted. First, this cohort study was conducted at a single center in China. Further inclusion and validation of results are required for multicenter samples from multiple provinces in China. Second, the study outcome was determined by qualitative results. If detailed quantitative data are available, it may be possible to analyze the quantitative relationship between the TyG index and carotid intima-media thickening or plaque progression. Third, participants were collected from general health checkups by using self-reported conditions, which lacked sufficiently objective medical records and medication information, such as hypoglycemic agents or lipid-lowering agents. However, in the general population of China, there is a relatively low proportion of individuals who take long-term stable levels of lipid-lowering and hypoglycemic agents.

## Conclusions

Our study demonstrated that participants with higher baseline and moderate-stable trajectory of the TyG index are independently associated with CAS progression. These findings support the fact that clinicians should closely monitor the TyG index during routine health examinations to recognize the development of carotid atherosclerotic plaques during prevention and treatment.

## Data Availability

The derived data that were generated in the current study are available from the corresponding author upon reasonable request.

## Abbreviations

TyG: Triglyceride-glucose
CVD: Cardiovascular disease
CAS: Carotid atherosclerosis
HR: Hazard ratio
CI: Confidence interval
SD: Standard deviation
TG: Triglycerides
FBG: Fasting blood glucose
HOMA-IR: Homeostatic model of insulin resistance
BMI: Body mass index
SBP: Systolic blood pressure
DBP: Diastolic blood pressure
TC: Total cholesterol
LDL-C: Low-density lipoprotein cholesterol
HDL-C: High-density lipoprotein cholesterol
cIMT: Carotid intima-media thickness
IQR: Interquartile range
LCTM: Latent class trajectory modeling
ASCVD: Atherosclerotic cardiovascular disease.
